# Novel hardware and concepts for unconventional computing

**DOI:** 10.1038/s41598-020-68834-1

**Published:** 2020-07-16

**Authors:** Martin Ziegler

**Affiliations:** Department of Microelectronic and Nanoelectronic Systems, TU Ilmenau, 98693 Ilmenau, Germany

**Keywords:** Nanoscience and technology, Materials science

## Abstract

Neuromorphic systems are currently experiencing a rapid upswing due to the fact that today's CMOS (complementary metal oxide silicon) based technologies are increasingly approaching their limits. In particular, for the area of machine learning, energy consumption of today's electronics is an important limitation, that also contributes toward the ever-increasing impact of digitalization on our climate. Thus, in order to better meet the special requirements of unconventional computing, new physical substrates for bio-inspired computing schemes are extensively exploited. The aim of this Guest Edited Collection is to provide a platform for interdisciplinary research along three main lines: memristive materials and devices, emulation of cellular learning (neurons and synapses), and unconventional computing and network schemes.

Neuromorphic engineering goes back to the 1980s to its inventor Carver Mead, who used the at this time relatively new VLSI (very-large-scale integration) technology to implement biologically inspired systems for information processing^1^. The idea was to find ways to transfer biological learning and memory processes to electronic circuits^2^. Today, this field has gained considerable new interest and expanded massively^3^. This development of the field is driven especially by improvements in the power efficiency of neuromorphic architectures, and the potential of new hardware, better tailored to meet the needs of machine learning^4^. An important contribution is made by non-volatile memory technologies (memristive technologies) that form the backbone of neuromorphic systems, since they allow us to mimic very precisely the learning and memory processes in hardware^3,4^. This Guest Edited Collection tries to bundle work in three main thematic areas of neuromorphic computing: memristive materials and devices, emulation of cellular forms of memory and learning, and neuromorphic computing (cf. Fig. 1). Since a continuous expansion of this collection is desired, the papers mentioned in this article can be considered a 'taster' for what is yet to come.

**Figure 1:**
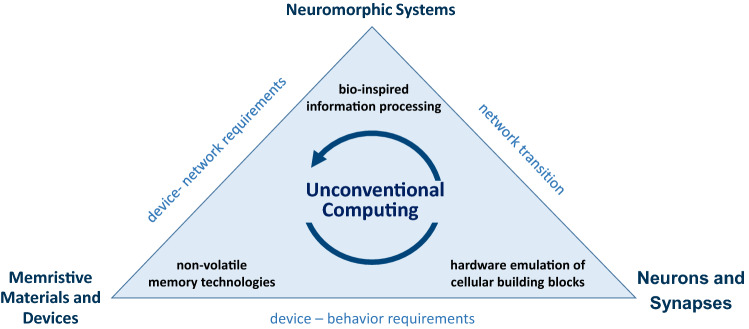
Thematic areas of unconventional computing: memristive materials and devices, emulation of cellular forms of memory and learning, neuromorphic computing. For the development of memristive devices, network requirements for the devices must be derived. Fundamental components of neural networks (neurons and synapses) must be reconstructed in such a way that they meet the special needs of memristive devices. Adequate models to emulate information processing on a local (cellular) level are required for a successful transition to complex system architectures.

Emulation of the central nervous system’s decentralized information processing, in which information is learned and stored locally, is the essential basis for bio-inspired computing^3^. This calls for novel memristive devices that are tailored to meet these specific requirements (cf. Fig. 1). In this respect, Vahl et al.^5^ investigate the nanoscale memristive properties of individual noble metal alloy nanoparticles that are sparsely encapsulated in a thin SiO_2_ dielectric matrix. They show evidence that alloy nanoparticles-based devices have reproducible diffusive switching characteristics and offer a high design versatility to tune such switching properties. The form of the resistance state is crucial for the successful training of artificial neural networks; Nikam et al.^6^ present a Li ion synaptic transistor with high ionic conductivity that allows linear conductance switching with several discrete non-volatile states. Also in^7^ the impact of Li-based devices for the emulation of synaptic behaviour is shown. The integration of cellular learning forms into the design of memristive components is shown by Zenya et al.^8^. Here, a 4-terminal device is presented that can simulate hetero-synaptic plasticity. Furthermore, Serb et al.^9^ were able to emulate the transmission and plasticity properties of real synapses by connecting memristive devices between brain and silicon spiking neurons. The aspect of biocompatibility is taken into account by investigating parylene-based memristive devices in Minnekhanov et al.^10^. Those devices can be used to emulate spike-timing-dependent plasticity (STDP) and based on this the authors implement a simple neuromorphic network model of classical conditioning.

For a successful transition, from the level of individual memristive devices to a multidimensional network level, the reconstruction of biological information processing by neurons and synapses is necessary^11^. This requires adapting the biological paradigms of information processing in such a way that they account for the special requirements of memristive devices^11^. In this respect, Ahmed et al.^12^ show how to implement different synaptic learning rules by utilizing a CMOS-compatible memristive approach, while Manicka et al. follow the question of how non-neural tissues could process information^13^. Stoliar et al.^14^ investigate the relationship between the STDP characteristics and spike types using ferroelectric-tunnel-junctions. In terms of neural functionalities, Rozenberg et al.^15^ introduce an ultra-compact leaky-integrate-and-fire neuron which has only three active devices but already emulates biologically realistic features. Furthermore, the authors claim that the ultimate ultra-compact limit can be reached by using materials which have a Mott transition. This advantage of Mott materials is used by del Valle et al.^16^ for emulating neural functionalities in the framework of a neuristor model.

It is the possibility of a parallel vector matrix multiplication within memristive crossbar arrays that enables energy-efficient in-memory computing, i.e. a decentralized computing in which information is processed and stored locally^17^. In this respect, Kingra et al.^18^ present a methodology to implement memory and logic operations simultaneously. Efficient hardware implementation of logic operation using memristive devices allows us to reduce the number of computation steps, as shown by Siemon et al.^19^. Kathmann et al.^20^ present a completely new methodology for logic operation which employs the heat flux exchanged in the near-field regime in nanoparticle networks. More biologically inspired approaches include supervised and unsupervised networks, oscillator systems, and stochastic computing. In this respect, Araujo et al.^21^ discuss the role of non-linear data processing on a speech recognition task, while Chou et al.^22^ report on an analogue computing system with coupled non-linear oscillators which is capable of solving complex combinatorial optimization problems. Nandakumar et al.^23^ demonstrate experimentally a supervised learning scheme using spiking neurons and phase change materials, while in^24,25^ photonic based-neuromorphic computing schemes are presented. For a significant reduction in power consumption, wake-up systems are of interest, which only switch on the more complex computing structures when they are needed. Gupta et al.^26^ shows an implementation of such a system using CMOS–MoS_2_ memtransistors.

The hope is that this Collection can give an overview of the complexity of the topic and show where the successes already achieved, but also the challenges of this topic, lie. Many thanks to all authors for their contributions to this Collection.
